# Phytochemical, acute toxicity and renal protective appraisal of *Ajuga parviflora* hydromethanolic leaf extract against CCl_4_ induced renal injury in rats

**DOI:** 10.1186/s12906-021-03360-9

**Published:** 2021-07-12

**Authors:** Samiullah Burki, Zeba Gul Burki, Muhammad Arif Asghar, Imdad Ali, Saba Zafar

**Affiliations:** 1 Department of Pharmacology, Institute of Pharmaceutical Sciences, Jinnah Sindh Medical University Karachi, Rafiqui H. J Shaheed Road, Karachi, 75510 Pakistan; 2 Federal Urdu University of Arts, Sciences and Technology, Karachi, Pakistan; 3 Department of Pharmaceutics, Institute of Pharmaceutical Sciences, Jinnah Sindh Medical University Karachi, Rafiqui H. J Shaheed Road, Karachi, 75510 Pakistan; 4 HEJ Research Institute of Chemistry, International Center for Chemical and Biological Sciences, University of Karachi, Karachi, Pakistan

**Keywords:** *Ajuga parviflora*, Antioxidant, Nephroprotective effect, Histopathology, DNA damages

## Abstract

**Background:**

Degenerative kidney diseases are mostly associated with oxidative stress. Natural products are considered as the antioxidants enrich food that can restrict the progress of oxidative stress induced disorders. Therefore, the present study was aimed to evaluate the renal protective effect of *Ajuga parviflora* leaf extract in carbon tetrachloride intoxicated rats.

**Methods:**

The hydromethanolic extract of *A. parviflora* leaves was obtained by extracting twice in 60% methanol. The principal bioactive constituents were detected by LC/MS analysis. Toxicity of plant extract was assessed using brine shrimp lethal toxicity test and acute toxicity model on healthy Sprague-Dawley male rats. Nephroprotective effects of plant extract were also evaluated on rats by inducing CCl_4_ renal toxicity in comparison with positive control and naïve groups. The dose of *A. parviflora* administered to animal was 100, 200 and 300 mg/kg. All administrations were given orally on an alternate day basis for 30 days. Urine and serum biomarkers were analyzed, along with antioxidant enzymes. Finally, the DNA damages, lipid peroxides, hydrogen peroxides and nitrites were assessed in rat’s renal tissue. The histopathology alterations in renal tissues were further studied for kidney damages.

**Results:**

The LC/MS analysis confirmed the presence of different important pharmacological compounds in *A. parviflora* methanolic leaf extract. The key bioactive compounds include pyocyanin, zonisamide, D Saccharic acid, altretamine, carbocyclic thromboxane A2, Sinapyl alcohol, and vitamin C. The important polypeptides identified include Lys-Tyr-Lys, His-His-Lys, Met-Asp-Arg, Phe-Val-Arg, and PyroGlu-Val-Arg. The LD_50_ of *A. parviflora* was found to be > 1000 μg/mL. *A. parviflora* administration significantly subsides CCl_4_ toxicity in rats, reduced the elevated level of RBCs, pus and epithelial cells. The abnormal elevated level of specific gravity, creatinine, urobilinogen, urea and albumin were also reduced to normal physiological level. The reduced urinary protein and pH were also normalized. The serum urobilinogen, urea and total bilirubin levels were also reversed to normal levels while the diminished albumin and total protein levels also came to normal. The important phase I and II enzyme levels were also reversed in *A. parviflora* administered rats. The H_2_O_2_, thiobarbituric acid reactive substance (TBARS) and nitrite levels were significantly decreased. Furthermore, the damaged DNA and histopathological changes in CCl_4_ exposed rats were also highly significantly reversed after the administration of *A. parviflora*. All effects were significant (*P < 0.05*) and highly significant (*P < 0.005*) at 100 and 300 mg/kg respectively.

**Conclusion:**

The restored urine and serum profile of various parameters to normal physiological levels suggests that the *A. parviflora* has potential antioxidant and repairing potential in renal disorders.

**Supplementary Information:**

The online version contains supplementary material available at 10.1186/s12906-021-03360-9.

## Background

Acute renal failure (ARF) is a leading cause of morbidity and mortality among kidney diseases in which abrupt or rapid decline in renal filtration function is observed. It is generally associated with a high ratio of mortality and has an independent effect on the risk of death. Globally, these complications depreciate normal renal physiology that leads to nitrogenous compounds retention and electrolytes imbalance in the body [1]. It is categorized into three major types i.e. intrinsic renal azotemia, prerenal azotemia and postrenal azotemia [2]. Recently, many studies reported the significant pharmacological activities of different plant extracts [3–7].

Free radicals such as reactive oxygen species are the agents that damage cellular function and cell organelles, mostly via disturbing oxygen-reduction balance [8]. Usually, the chemicals like CCl_4_, polycyclic hydrocarbons and drugs such as acetaminophen are the agents that are commonly responsible for nephrotoxicity in humans and animals. CCl_4_ induces toxicity in various organs of animals via the production of oxygen free radicals. The generated free radicals further damage the cell membrane through the lipid peroxidation process. Antioxidants are the agents to scavenge such chain reactions due to free radicals and prevent organ injury. Naturally, there are endogenous antioxidants like superoxide dismutase, glutathione-s-transferase, glutathione peroxidase, catalase and glutathione to cope with the free radicals [9].

*Ajuga parviflora* (*A. parviflora*) belong to *Ajuga* genous and *Lamiaceae* family found in the hilly region of Pakistan and Afghanistan. The plant traditionally has been reported for the cure of asthma, fever, hepatitis C virus (HCV), jaundice, arthritis, cancer and wound. It is also used locally for the treatment of various stresses induces disorders. The plant has also been previously reported for the treatment of eye allergic irritation, insects biting poison, stomach ache and hepatic impairments [10]. However, the nephroprotective activity of *A. parviflora* has not been previously explored. Previously, many of the herbal compounds isolated from the essential oil of plant extract were reported for the treatment of ARF. For instance, thymoquinone, ginsenosides, gingerol, proanthocyanidin-BP1, silymarin, crocin, quercetin and various organosulfur are the major compounds reported for ARF management [11, 12]. Most of these phytochemicals with known hepatoprotective activities have been previously identified in *A. parviflora* extract [10, 13]. However, the *A. parviflora* extract and its pure compounds have not been investigated for the nephroprotective activity against CCl_4_ induced toxicity. Therefore, we aim to study the nephroprotective effects of *A. parviflora* extract against CCl_4_ induced toxicity on molecular level. In addition, *A. parviflora* was reported for potent hepatoprotective effect in our previous study [14]. However, the hepatoprotective and nephroprotective effects are commonly associated with each other in previous studies since both are attained via protection from free radicals [15, 16]. In addition, this plant has also been reported for important antioxidant (free radicals scavenging) compounds like withanolides and alkaloids [17]. Due to this correlation and also the reported promising pharmacological properties and antioxidant compounds of *A. parviflora*, the current study was aimed to assess the acute toxicity and nephroprotective effect of *A. parviflora* on CCl_4_ induced renal protective activity*.*

## Methods

### Plant material

Fresh leaves of *Ajuga parviflora* (L.) were collected from 2 year old plant in the month of June-2019 from the hilly region of South Waziristan, Khyber Pakhtunkhwa (KPK), Pakistan with the accordance of relevant institutional, national, and international guidelines and legislation after authentication through its local name trakha boti. Further, the plant was identified by pharmacognosist at the National Agriculture Research Center (NARC) Islamabad and the voucher specimen (RAW 101001A) was submitted at the NARC. The voucher of the specimen was also submitted to the herbarium library of the Faculty of Pharmacy, Federal Urdu University of Arts Science and Technology (FUUAST).

### Extraction

The plant leaves after the collection were dried under shade and crushed to a fine powder. About 1.2 kg powder was macerated in 60% methanol (Sigma Aldrich, USA) for 15 days. After 15 days the plant extract was further filtered via Whatman filter paper. After filtration, the extract was concentrated using a rotary evaporator (B-490, Buchi, Switzerland) at 45 °C and obtained 122 g greenish *A. parviflora* hydromethanolic (APHM) leaves extract [18, 19]. The extract was stored at 4 °C for further studies.

### Liquid chromatography–mass spectrometry (LC/MS)

The bioactive fraction of *A. parviflora* was further subjected to phytochemical analysis. The analysis was carried out on (Agilent Maxis II HD Q-TOF 6530) with positive ionization and HPLC inlet modes with MS_2_ spectrum type. The HPLC column was a reverse-phase Acclaim RSLC, C18, 50 mm × 2.1 mm, 2.2 μm, 120 ^O^A (Thermoscientific). The solution of the test sample for LCMS was prepared in methanol as mobile phase (Sigma Aldrich, USA) and the instrumental condition was maintained at 280 °C, while the sample flow rate was set at 8 μL/min. An amount of 20 μL was set as injected volume for sample. The mass range was selected from 50 to 1000 m/z as reported by Khan et al. [20]. The obtained spectrum was matched with NIST/ LCMS library for the identification of important compounds.

### Brine shrimp lethality for LD_50_ estimation

Seawater (36–38 g/L sea salt, 7.4 pH) was arranged from the local seashore. The seawater was filled in a rectangular dish (22 × 32 cm) and 50 mg brine shrimp eggs freshly taken were sprinkled in water followed by incubation for two days at 37 °C. After hatching, mature nauplii (10 larvae/ vial) were treated with the prepared leaf extracts of *A. parviflora* at the concentrations of 10, 100, 1000 and 10,000 μg/mL in dimethyl sulfoxide (DMSO). However, different concentrations of DMSO (Sigma Aldrich, USA) and etoposide (Sigma Aldrich, USA) were taken as negative control and standard drug respectively. Percent lethality for the samples was calculated according to the method mentioned by Asghar et al. [21]. Briefly, a volume of 1 mL aliquot from each tested concentration was taken in vial and dried for 24 h at ambient temperature. Twenty napulii with 10 mL sea water were added in each vial and kept under illumination at 25 °C. After 24 h the live shrimps were counted using a magnifying glass. However, lethal concentrations (LC_50_) were calculated using linear regression analysis while toxicity profiles were evaluated according to Clarkson’s toxicity criterion, i.e. LC50 > 1000 μg/mL, 500–1000 μg/mL, 100–500 μg/mL, 0–100 μg/mL were categorized as non-toxic, low toxic, medium toxic and highly toxic respectively [22]. Each test concentration was assessed three times.

### Animal’s treatment

The Sprague Dawley rats, of average weight about 150–200 g were purchased from DOW University of Health Sciences (DUHS). The consent form was filled and submitted to the DUHS animal house in-charge for the used of animals in this study. Animals were kept in suitable plastic cages with a prerequisite temperature of 23 ± 2 °C with humidity 50–60% in a light-dark cycle of 12 h each during the whole study. Both control and tested animals were kept in the same environment and provided with the standard food. All studies were conducted following the National Institute of Health (NIH) guide for the care and use of Laboratory Animals and in compliance with the ARRIVE guidelines [23]. The animals were freely allowed access to food and water. For animal studies, an ethical approval (AP-SM-20B) was obtained from the institutional review board of Federal Urdu University, Karachi. At the end of the study, the animals were euthanized using the cervical dislocation method with all possible effort being given to minimize suffering. Before euthanasia, each animal was sedated with an intravenous injection of medetomidine (Sigma Aldrich, USA) in a concentration of 2 μg/kg [24].

### Acute toxicity study

Rats were randomly selected and divided into six groups with three rats in each group. The animals were kept fasted only allowed to water for at least 7 h. The extract solutions were prepared in 0.9% normal saline. Animals of group 1–6 received *A. parviflora* solution orally at dose of 100, 500, 1000, 2000, 3000 and 5000 mg/kg respectively, while group 7 received normal saline and considered as a control group. At the end of the 14-day study period, animals were euthanized and their organs including heart, liver, kidney and stomach were excised, weight and compared their morphologyies with the control animals group [25].

### CCl_4_ induced renal toxicity study

Thirty-six rats were divided into six groups (six animals in each group). Group A animals were considered as naïve group (received no treatment) while animals in remaining groups received 1 mL/kg body weight CCl_4_ (Sigma Aldrich, USA) intraperitoneally in 30% v/v olive oil for induction of renal toxicity [26]. However, Group B was considered as positive control (received CCl_4_ only) while Group C was treated with Silymarin (Sigma Aldrich, USA) oral solution at a dose of 100 mg/kg as standard treatment. Groups D, E and F were treated orally with *A. parviflora* with the dosing of 100, 200 and 300 mg/kg respectively as test solutions. All administrations were given on an alternate day basis (1 day gap) for 30 days. After the last treatment, the urine samples were collected from animals using metabolic cages and stored at − 70 °C for further analysis. The animals were euthanized after application of medetomidine in a concentration of 2 μg/kg (IV) and blood samples were collected by direct cardiac puncture. The blood samples were centrifuged at 4 °C, 500×g for 15 min to collect sera for biochemical estimation. The kidney of each animal was excised, washed with ice-cold water for removal of debris and stored in liquid nitrogen at − 70 °C for homogenate tests. The small piece from each kidney was isolated and stored in 10% formalin (Sigma Aldrich, USA) for histopathological evaluation. The further histopathological activity of *A. parviflora* was performed as previously reported by GC/MS assisted phytochemical analysis of *A. parviflora* leaf extract along with anti-hepatotoxic effect against anti-tubercular drug-induced liver toxicity in rat [14].

### Urine analysis

Urine samples were collected for analysis of urine pH, specific gravity, albumin level, red blood cells, pus cells and epithelial cells. Further, the urine samples were also evaluated for biochemical analysis i.e. creatinine urobilinogen, urea, protein and albumin The tests were performed using standard diagnostic kits (AMP Krenngasse 12, 1810 Graz, Australia) as instructed by the manufacturer.

### Serum analysis

Serum analysis was performed to the analyzed levels of creatinine urobilinogen, urea, total bilirubin, total protein and albumin by diagnostic kit (AMP Krenngasse 12, 1810 Graz, Australia) as per kit manufacturing guidelines and instruction.

### Protein estimation

In renal tissue soluble protein was estimated according to the commonly used method reported by Lowry et al. [27]. Since this method comprised of different reagents; Reagent A, is 2% Na_2_CO_3_ in 0.10 N NaOH. Reagent B, is 0.5% CuSO_4_.5H_2_0 in 1% sodium tartrate while Reagent C composed of alkaline copper solution. Briefly, 0.1 mL of supernatant was mixed with 1 mL of the alkaline copper solution (Reagent C) followed by thorough mixing for 10 min at room temperature. Then 0.10 mL of diluted Folin reagent was added rapidly and the mixture was incubated for 30 min. The mixture was subjected to the determination of optical density at 595 nm. Soluble proteins were calculated in renal tissue using a standard curve of bovine serum albumin.

### Assessment of antioxidant enzymes in renal tissues

Antioxidant enzymes in renal tissue were determined by homogenizing the renal tissue. The 10x homogenate was prepared by adding potassium phosphate buffer (pH 7.4). The homogenate was further centrifuge at 1500 g for 12 min at 40 °C. The supernatant of centrifuged was used for the quantification of various antioxidant enzymes.

#### Catalase (CAT) activity

A mixture of 100 μL of 5.9 mM H_2_O_2_ (Sigma Aldrich, USA) and 625 μL of 50 mM potassium phosphate buffer (Sigma Aldrich, USA) (pH 5.0) was prepared for the determination of catalase activity in renal tissue. To this mixture, 25 μL of supernatant was added. The catalase present in the supernatant was disintegrated to H_2_O_2_ which will eventually cause declined in its concentration in the mixture. The level of catalase activity was monitored via the decline in absorbance at 240 nm for 1 min. The change in absorbance by 0.01 as units/min reflects the activity of 1 unit of catalase [28].

#### Peroxidase (POD) activity

The peroxidase activity of plant extract was determined according to the method described by Chance et al. with slight modifications [28]. Briefly for the preparation of the reaction mixture, about 25 μL (20 mM), 75 μL (40 mM) and 625 μL (50 mM) of guaiacol (Sigma Aldrich, USA), H_2_O_2_ and potassium phosphate buffer (pH 5) were mixed respectively. A volume of 25 μL supernatant of renal tissue was added to the reaction mixture and absorbance was recorded at 470 nm for 1 min. A 0.01 unit/min changed was considered to be the peroxidase activity of plant extract.

#### Superoxide dismutase (SOD) activity

The reaction mixture was prepared in such a way that phenazine methosulphate (Sigma Aldrich, USA) (186 μM) and sodium phosphate buffer (Sigma Aldrich, USA) (0.052 mM) were mixed in a volume of 50 μL and 600 μL respectively. The renal supernatant was prepared by centrifuging the homogenate at 1500 g for 10 min, and then further for 10,000 g for 15 min. This supernatant was added to the previously prepared reaction mixture followed by the addition of 100 μL NADH (780 μM). After 1 min, the reaction was stopped by the addition of 500 μL glacial acetic acid (Sigma Aldrich, USA). The optical density of chromogen was quantitatively estimated at 560 nm on a spectrophotometer, and enzyme activity was calculated such that the enzyme concentration required for inhibiting 50% chromogen in 1 min [29].

#### Glutathione-S-transferase (GST) activity

The conjugation between reduced glutathione (GSH) and 1-chloro,2,4-dinitrobenzene (CDNB) is the basic phenomena behind glutathione-s-transferase activity [30]. Initially, sodium phosphate buffer (pH 6.5, 720 μL) and CDNB (1 mM, 12.5 μL) were mixed. This mixture was incubated for 10 min at 37 °C. About 150 μL renal tissues supernatant was added to the prepared mixture. To this solution, 100 μL of glutathione (1 mM) was added for initiation of reaction and the elevation of absorbance was measured at 340 nm for 10 min. However, a similar procedure was followed for blank sample except for renal tissue supernatant.

#### Glutathione reductase (GSR) activity

The GSR activity was performed by treating the 50 μL of renal supernatant with the reaction mixture of 25 μL oxidized glutathione (1 mM), 50 μL of EDTA (Sigma Aldrich, USA) (0.1 mM) and 825 μL sodium phosphate buffer (0.1 M, pH 7.6) as reported by Shareen et al. [31]. Then, 50 μL of NADPH (0.1 mM) was added to start the reaction. The declined in optical density at 340 nm was observed for 20 min.

### Estimation of biomolecules in renal tissue

#### Reduced glutathione (GSH) assay

To determine the glutathione in renal tissue, the glutathione was oxidized from 5,5′-diathio-bis-(2-nitrobenzoic acid) to a yellow 5′-thio-2-nitrobenzoic acid using an oxidizing agent i.e. sulfahydral reagent. Initially, 500 μL renal supernatant was added to 4% sulfosalicylic acid to obtained precipitate. This mixture was allowed to incubate at 4 °C for at least an hour followed by centrifugation for 20 min at 1200 g to obtained supernatant. About 33 μL supernatant was mixed with potassium phosphate buffer (900 μL, 0.1 M, pH; 7.4) and 66 μL of 100 mM 5,5′-diathio-bis-(2-nitrobenzoic acid). The yellow color complex was obtained and the optical density of this complex was measured at 412 nm. The μM of GSH/g of renal tissue was determined [32].

#### Lipid peroxidation (TBARS) assay

The lipid peroxidation assay was performed following the method explained by Iqbal et al. with some modifications [33]. The assay technique was principally comprised of thiobarbituric acid reactive substance (TBARS) measurement in the renal tissues. The assay was carried out by mixing 100 μL of renal tissue with 10 μL FeCl_3_ (100 mM), 100 μL of ascorbic acid (100 mM) and 290 μL of phosphate buffer (pH 7.4) followed by incubation at 37 °C for 1 h. The reaction was stopped by the addition of 10% trichloroethanoic acid (TCA) in a volume of 500 μL and the tubes were placed in a boiling water bath for 15 min. Then tubes were cooled for 5 min in crushed ice followed by centrifugation at 2500 g for 10 min. The amount of TBARS was measured at 535 nm and the TBARS activity was indicated as TBARS formed as min/mg tissue.

#### Hydrogen peroxide (H_2_O_2_) assay

In the procedure of this assay, the phenol was oxidized to hydrogen peroxide (H_2_O_2_) mediated by peroxidase enzymes. The process was started with the preparation of the reaction mixture in the composition of 1 mL phenol red (0.28 nM), 2 mL of renal tissue homogenate, dextrose (5.5 nM) and phosphate buffer (0.05 mM, pH 7.0). To this solution, horse red peroxidase (8.5 units) was added for initiation of reaction and the mixture was allowed to incubate for 1 h at 37 °C. The reaction was terminated by the addition of 0.01 mL NaOH (10 N). The resultant product was centrifuged for 15–20 min at 800 g. The absorbance of the test sample was noted at 610 nm [34].

#### Nitrite assay

In the renal tissue, the concentration level of nitrite was carried out as explained by Green et al. [35]. The homogenate of renal tissue was treated with equal volumes of NaOH (0.3 M) and 5% of ZnSO_4_. Further, the mixture was centrifuged for 15–20 min at 6400 g to get protein-free supernatant. The optical density of the supernatant was assessed at 540 nm by reacting with 20 μL of Griess reagent which was used as a blank.

### Histopathological study

The animals were euthanized and the kidney of different group’s rats was subjected to fixation process followed by dehydration in alcohol (90%). The tissue of the kidney was further processed according to the method described in our previous study [14]. Finally, the thin slice of kidney tissue (3–4 μm) was subjected to the removal of wax and then stained with hematoxyline-eosin (Sigma Aldrich, USA).

### RT-PCR Telomeric repeat amplification protocol (TRAP) assay

Telomerase activity was carried out as explained by Wen et al. in their previous study with slight modifications [36]. Briefly, about 100 mg sample was taken from the isolated kidney and washed with an ice-cold aqueous mixture of KOH (10 mmol, pH 7.5), MgCl_2_ (1.5 mmol), KCl (10 mM), dithiothreitol (1 mM) and 20 μL of RNAs inhibitors. Then, the sample was homogenized in 200 μL of the ice-cold lysis buffer system. The homogenate product was subjected to an incubation process for 30 min on ice and then further centrifuged at 10000 g for 30 min at 4 °C.

A 50 μL PCR reaction mixture comprised of 38.6 μL of Diethyl pyrocarbonate (DEPC) treated water, 2 μL of protein extract (6 μg), 5 μL 10x TRAP reaction solution, 2 μL each dNTP (50 μmol), 0.4 μL Taq DNA polymerase (2 U) and 2 μL of TS primer sequence (5′- AATCCGTCGAGCAGAGTT-3′). The mixture of PCR-reaction was further incubated in 25 °C on a water bath for 30 min to extend the TS primer. A volume of 2 μL of CX primer (0.1 μg) with a sequence of 5′-CCCTTACCCTTACCCTTACCCTTAA-3′ was added to the reaction mixture. The whole mixture was again processed in PCR cycles at various temperatures. Finally, the temperature was set at 72 °C for 1.5 h. Once the amplification was completed, a combination dye of xylenocyanol and bromophenol blue along with 50% glycerol was loaded to each PCR product. About 25 μL of each sample was loaded on non-denaturing polyacrylamide gel (12.5%). In a final step, the gel was fixed in a solution of acetic acid (0.5%) and ethanol (10%). The obtained product was stained for 10 min with 0.2% of AgNO_3_ and allowed to incubate for 15 min in an incubation chamber containing a solution of formaldehyde (0.1%) and NaOH (3%). The photographs were then captured for further study.

### Statistical analysis

The data were expressed as mean ± SEM. One-way ANOVA of variance was applied using a Graph pad for inferential analysis on obtained results. For multiple comparisons among the various treatment groups, a Tukey *posthoc* test was also utilized. The results were considered as significant at *p* < 0.05 and highly significant at *p* < 0.005.

## Results

**Fig. 1:**
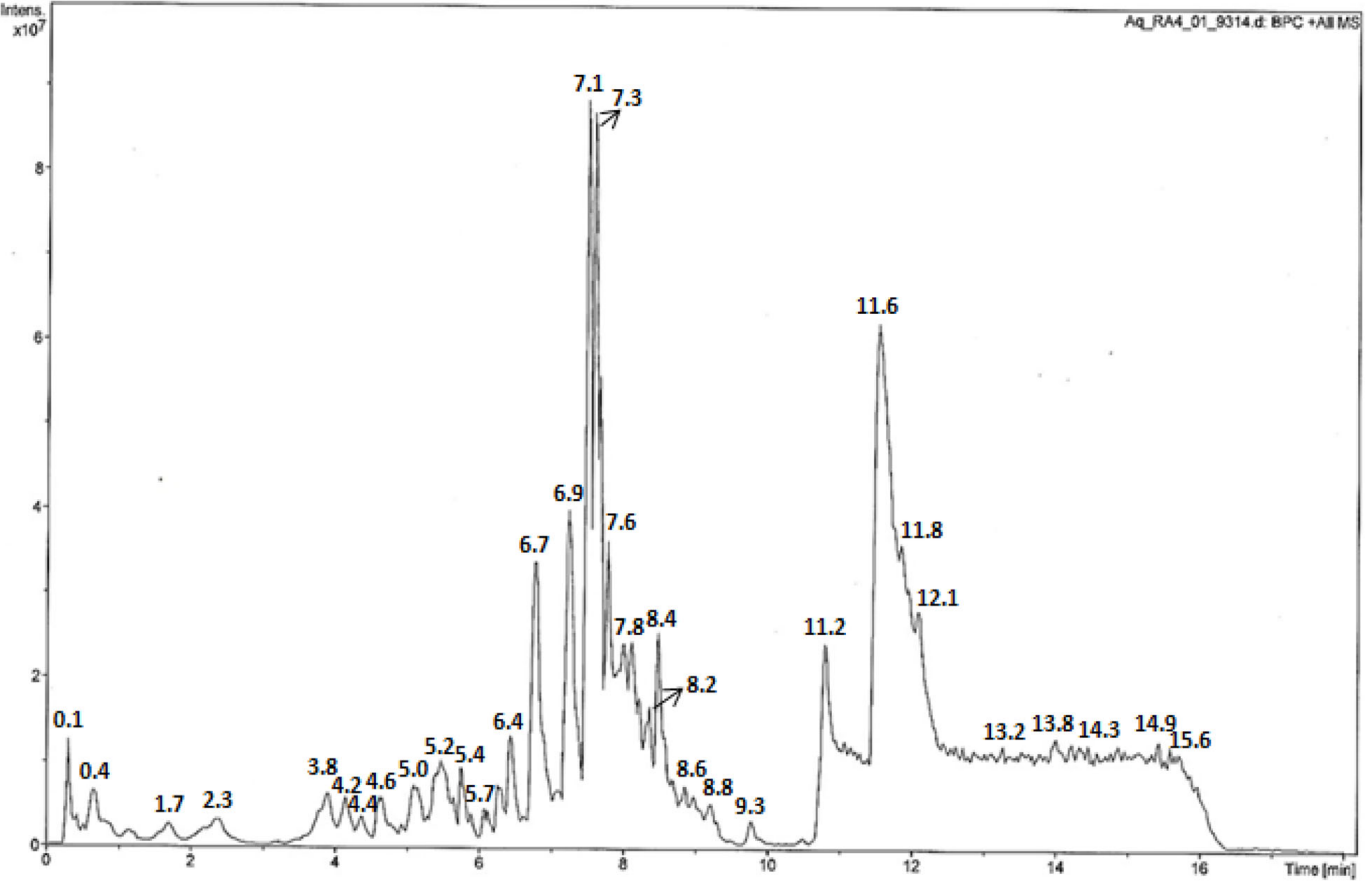
LCMS spectra of *A. parviflora*

Phytochemical analysis of *A. parviflora* leaves extract was carried out on LC/MS technique. The obtained spectrum was compared with the NIST library, which revealed the presence of important biochemical constituents such as, 9-Ethyl-9H-carbazol 3-ylamine, (2R,3R)-(−) 2-benzyloxy 1,3,4- butanetriol, pyocyanin, zonisamide, D Saccharic acid, altretamine, carbocyclic thromboxane A2, Sinapyl alcohol, and vitamin C. The important organic polypeptides were also identified i.e. Lys-Tyr-Lys, His-His-Lys, Met-Asp-Arg, Phe-Val-Arg, and PyroGlu-Val-Arg **(**Fig. 1**)** and Table 1.

**Table 1:** Retention time (Rt) for qualitative confirmation of the compound occurring in *A. parviflora* hydroalcoholic extracts by LC/MS

S.no	Compound	Rt (min)
1	9-Ethyl-9H-carbazol 3-ylamine	0.1
2	(2R,3R)-(−) 2-benzyloxy 1,3,4- butanetriol	0.4
3	Pyocyanin	1.7
4	Zonisamide	2.3
5	D Saccharic acid	3.8
6	Altretamine	6.9
7	Carbocyclic thromboxane A2	7.3
8	Sinapyl alcohol	7.6
9	Lys-Tyr-Lys	7.8
10	His-His-Lys	11.6
11	Met-Asp-Arg	12.1
12	Phe-Val-Arg	13.2
13	PyroGlu-Val-Arg	13.8

### Brine shrimp’s lethality

For toxicological profile, the *A. parviflora* hydromethanolic extract was tested against brine shrimps nauplii. The results in Table 2 suggested that the *A. parviflora* extract did not show the lethal effect at 1000 μg/mL and the LD_50_ of *A. parviflora* was found to be 2853.1 μg/mL.

**Table 2:** Toxicity profiles of APHM test samples using brine shrimp cytotoxicity test

Groups	Dose μg/mL	Survivors (%)	LC_**50**_^c^ μg/mL	Toxicity profile
**DMSO**^d^ **(v/v)**	1000	100	–	Non-toxic
	5000	100		
	10,000	100		
	25,000	100		
**APHM**^e^	10	100	2853.1	Non-toxic
	100	82 ± 7.64		
	1000	71.4 ± 9.33^a^		
	10,000	22.1 ± 6.24^b^		
**Etoposide**	10	19.4 ± 4.25^b^	8.4	Toxic

Clarkson criteria: LC_50_ > 1000 μg/mL are non-toxic, LC_50_ of 500–1000 μg/mL are low toxic, LC_50_ of 100–500 μg/mL are medium toxic, LC_50_ of 0–100 μg/mL are highly toxic

^a^*p* ≤ 0.05 significant as compared to control, ^b^*p* ≤ 0.005 highly significant as compared to DMSO

^c^Lethal concentration, ^d^Dimethyl sulfoxide, ^e^*Ajuga parviflora* hydromethanolic extract

### Acute toxicity study in rats

Overall animal health evaluation such as average weight variation, skin ulceration, loss of activity, diarrhea, hematuria, salivation, tremor, vomiting, edema and aggressive behavior were carried out and no changes were found in all control and test groups before and during the total period of the experiment.

Similar to brine shrimp toxicity results, the acute toxicity study outcomes suggested that the extract of *A. parviflora* was safe at all prescribed concentrations while slight hair loss was observed in 30% of animals at the dose of 5000 mg/kg.

### Effect of APHM on physical properties of urine in rat

The levels of urine markers in the *A. parviflora* administered groups in comparison with control and standard Silymarin administered groups are given in Table 3. The results showed that the urine of the control group was significantly (*p < 0.05*) and highly significantly (*p < 0.005*) moved to acidic pH as compared to standard and *A. parviflora* treated rats in dose dependent manner. In addition, the urine specific gravity of the standard and *A. parviflora* (300 mg/kg) treated group was also decreased in a highly significant (*p < 0.005*) manner as compared to control animals.

**Table 3:** Effect of APHM on physical properties of urine after CCl_4_ induced renal injury in rats

Treatment Groups	Doses (mg/kg)	pH	Specific gravity	RBC	Pus cell	Epithelial cell
**Naïve**	–	7.22 ± 0.13^b^	1.08 ± 0.08^b^	1.10 ± 0.21^b^	2.31 ± 0.21^b^	1.55 ± 0.45^b^
**Control**	–	6.15 ± 0.21	1.41 ± 0.05	7.50 ± 1.10	14.45 ± 1.15	19.15 ± 1.16
**APHM**^c^	100	6.55 ± 0.11^a^	1.15 ± 0.10^a^	3.08 ± 0.53^a^	4.85 ± 0.25^b^	5.58 ± 0.65^b^
**APHM**	200	6.85 ± 0.12^a^	1.03 ± 0.01^b^	1.55 ± 0.11^b^	2.35 ± 0.55^b^	2.88 ± 0.35^b^
**APHM**	300	7.04 ± 0.20^b^	1.05 ± 0.02^b^	1.11 ± 0.21^b^	2.45 ± 0.54^b^	1.44 ± 0.22^b^
**Silymarin**	100	7.12 ± 0.12^b^	1.01 ± 0.05^b^	2.0 ± 0.50^b^	2.75 ± 0.75^b^	4.56 ± 0.87^b^

Naïve groups did not received any treatment while control received CCl_4_ only

*n* = 6, Average values ± SEM,

^a^*p* ≤ 0.05 significant as compared to control, ^b^*p* ≤ 0.005 highly significant as compared to control

^c^*Ajuga parviflora* hydromethanolic extract

### Effect of APHM on biochemical markers in rat urine

The biochemical markers in rat urine samples such as creatinine, albumin, urea, urobilinogen and proteins were estimated against control and *A. parviflora* leaf extract **(**Table 4**)**. At a dose of 100 mg/kg, *A. parviflora* showed significant (*p < 0.05*) attenuation of creatinine, albumin, urea and urobilinogen while it raised the level of total proteins in a significant manner (*p < 0.05*) as compared to the control group. The groups treated with 200 mg/kg *A. parviflora* leaf extract higher significantly (*p < 0.005*) reduced the creatinine, urea and urobilinogen while it raised the level of total protein. The administration of *A. parviflora* leaf extract at a higher dose i.e. 300 mg/kg elevated the total urinary proteins while it decreased the aforesaid biochemical parameters such as creatinine, albumin, urea and urobilinogen noticeably (*p < 0.005*). The standard group also restored the renal biochemical markers closer to the naïve group.

**Table 4:** Effect of APHM on biochemical markers of urine after CCl_4_ induced renal injury in rats

Treatment Groups	Doses (mg/kg)	Creatinine (mg/dL)	Urobilinogen (mg/dL)	Urea (mg/dL)	Albumin (mg/dL)	Protein (mg/dL)
**Naïve**	–	2.74 ± 0.24^b^	4.38 ± 0.28^b^	103.22 ± 4.55^b^	4.56 ± 0.44^b^	38.47 ± 1.43^b^
**Control**	–	5.11 ± 0.65	32.20 ± 2.12	133.55 ± 6.12	18.16 ± 0.85	22.95 ± 1.39
**APHM**^c^	100	3.45 ± 0.12^a^	22.25 ± 1.12^a^	119.34 ± 3.45^a^	11.82 ± 0.34^a^	27.53 ± 1.70
**APHM**	200	2.34 ± 0.21^b^	13.62 ± 0.57^b^	110.76 ± 3.32^b^	7.42 ± 0.35^b^	33.82 ± 1.72^a^
**APHM**	300	2.56 ± 0.16^b^	5.04 ± 0.25^b^	104.14 ± 2.85^b^	5.08 ± 0.12^b^	40.14 ± 1.22^b^
**Silymarin**	100	2.61 ± 0.20^b^	10.15 ± 0.85^b^	105.58 ± 2.46^b^	6.42 ± 0.22^b^	35.77 ± 1.44^b^

Naïve groups did not received any treatment while control received CCl_4_ only

*n* = 6, Average values ± SEM,

^a^*p* ≤ 0.05 significant as compared to control, ^b^*p* ≤ 0.005 highly significant as compared to control

^c^*Ajuga parviflora* hydromethanolic extract

### Effect of APHM on biochemical markers in rat serum

The biochemical markers such as creatinine, albumin, urea, urobilinogen and proteins in rat serum samples were estimated in control and *A. parviflora* leaves extract (Table 5). The groups treated with *A. parviflora* at 100 mg/kg showed significant (*p < 0.05*) reduction in creatinine, albumin, urea and urobilinogen while it increased the level of total proteins. At 200 mg/kg of *A. parviflora* leaf extract, a significant (*p < 0.05*) reduction in the levels of creatinine, urea and urobilinogen were observed while it raised the level of serum albumin and total protein in a significant manner (*p < 0.05*). However, at a higher dose of *A. parviflora* leaf extract (300 mg/kg) it elevated the total serum proteins and reduced the aforementioned biochemical parameters in a highly significant manner (*p < 0.005*).

**Table 5:** Effect of APHM on biochemical markers of serum after CCl_4_ induced renal injury in rats

Treatment Groups	Doses (mg/kg)	Creatinine (mg/dL)	Urobilinogen (mg/dL)	Urea (mg/dL)	Albumin (mg/dL)	Protein (mg/dL)
**Naïve**	–	0.75 ± 0.04^b^	13.55 ± 1.20^b^	26.55 ± 1.29^b^	5.22 ± 0.16^b^	8.55 ± 0.44^b^
**Control**	–	1.85 ± 0.25	31.58 ± 1.53	73.15 ± 3.51	2.47 ± 0.15	5.15 ± 0.41
**APHM**^c^	100	1.35 ± 0.12^a^	21.13 ± 1.44^a^	38.14 ± 1.36^a^	3.85 ± 0.35^a^	6.77 ± 0.35^a^
**APHM**	200	1.06 ± 0.03^a^	17.56 ± 0.84^b^	29.85 ± 1.64^b^	4.42 ± 0.14^a^	7.53 ± 0.15^a^
**APHM**	300	0.68 ± 0.02^b^	14.05 ± 0.82^b^	25.75 ± 1.55^b^	5.05 ± 0.33^b^	8.45 ± 0.26^b^
**Silymarin**	100	0.97 ± 0.03^a^	18.88 ± 1.65^b^	30.00 ± 1.48^b^	4.41 ± 0.21^a^	7.64 ± 0.32^a^

Naïve groups did not received any treatment while control received CCl_4_ only

*n* = 6, Average values ± SEM,

^a^*p* ≤ 0.05 significant as compared to control, ^b^*p* ≤ 0.005 highly significant as compared to control

^c^*Ajuga parviflora* hydromethanolic extract

### Effect of APHM on phase I antioxidant and phase II enzymes in renal tissue of rat

The enzymes such as catalase, peroxidase and superoxide dismutase indicated in (Table 6) revealed significant (*p < 0.05*) improvement in their levels after administration of *A. parviflora* at 100 mg/kg compared to control animals. The enzyme improvement was observed in dose dependent manner. At 200 mg/kg dose of *A. parviflora*, animals showed similar alleviation of enzymes observed at 100 mg/kg *A. parviflora*. A progressive improvement of damaged renal tissue in a highly significant (*P < 0.005*) manner was noted at 300 mg/kg dose of *A. parviflora*.

**Table 6:** Effect of APHM on phase I and phase II antioxidant enzymes in renal tissue after CCl_4_ induced renal injury in rats

Treatment groups	Doses (mg/kg)	Phase I antioxidant enzymes			Phase II antioxidant enzymes	
		Catalase U/min	POD^c^ U/min	SOD^d^ U/min	GST^e^ (nM/min/mg protein)	GSR^f^(μg/mg protein)
**Naïve**	–	5.75 ± 0.28^b^	6.72 ± 0.36^b^	5.65 ± 0.22^b^	22.62 ± 1.21^b^	138.44 ± 3.45^b^
**Control**	–	2.32 ± 0.21	3.10 ± 0.23	1.85 ± 0.16	10.58 ± 0.35	71.25 ± 2.57
**APHM**^g^	100	3.25 ± 0.25	4.65 ± 0.35	3.12 ± 0.15^a^	20.25 ± 1.00^b^	109.20 ± 2.17^a^
**APHM**	200	4.50 ± 0.22^a^	5.95 ± 0.23^a^	4.95 ± 0.44^b^	21.11 ± 1.10^b^	127.25 ± 2.55^b^
**APHM**	300	5.78 ± 0.41^b^	6.68 ± 0.1b^a^	5.56 ± 0.52^b^	21.82 ± 1.16^b^	139.12 ± 1.82^b^
**Silymarin**	100	5.65 ± 0.11^b^	5.70 ± 0.45^b^	5.22 ± 0.33^b^	21.45 ± 1.15^b^	131.21 ± 2.65^b^

Naïve groups did not received any treatment while control received CCl_4_ only

*n* = 6, Average values ± SEM,

^a^*p* ≤ 0.05 significant as compared to control, ^b^*p* ≤ 0.005 highly significant as compared to control

^c^Peroxidase, ^d^Superoxide dismutase, ^e^Glutathione-S-transferase, ^f^Glutathione reductase, ^g^*Ajuga parviflora* hydromethanolic extract

The phase II enzymes are involved in the biotransformation of xenobiotics and endobiotic that include carcinogen metabolism. These enzymes include glutathione-S-transferase (GST), glutathione reductase (GSR) indicated in (Table 6). The GST at all doses of plant extract showed a similar pattern of improvement in dose dependent manner. GSR was highly significantly (*p < 0.005*) improved at the doses of 200 and 300 mg/kg. The results of *A. parviflora* leaf extract were very much similar to naïve and standard drug Silymarin.

### Effect of APHM on the biomolecules of renal tissues

In CCl_4_ treated experimental animals, stress is considered as the key factor in kidney injury. Aside from the toxicities of CCl_4_ on phase I and phase II antioxidant enzymes; changes in concentration of other biochemical molecules have been indicated in Table 7. The renal tissue biomarker GSH has increased significantly after administration of *A. parviflora* in dose dependent manner. The lipid peroxidase (TBARS) assay results were also changed in CCl_4_ induced intoxicated animals (*p < 0.05*) as compared to naïve animals. However, 100 mg/kg of plant extract revert the harmful elevated level of TBARS at (*p < 0.05*). Similarly, 200 and 300 mg/kg also attenuated the level of TBARS at (*p < 0.005*). The results were also statistically very much similar to standard Silymarin administered animals. After administration of *A. parviflora,* reduction in the elevated H_2_O_2_ and nitrite levels in a significant and dose dependent manner was observed.

**Table 7:** Effect of APHM on the biomolecules of renal tissues after CCl_4_ induced renal injury in rats

Treatment Groups	Doses (mg/kg)	GSH^c^ μM/g tissue	TBARS^d^(nM/min/mg protein)	H_**2**_O_**2**_ μM/min	Nitrite μM/min
**Naïve**	–	30.57 ± 2.20^b^	3.18 ± 0.62^b^	1.22 ± 0.17^b^	108.45 ± 3.15^b^
**Control**	–	16.35 ± 2.22	5.88 ± 0.45	2.85 ± 0.26	145.35 ± 2.20
**APHM**^e^	100	24.65 ± 1.45^a^	4.04 ± 0.20^a^	2.15 ± 0.21^a^	119.25 ± 4.55^a^
**APHM**	200	29.33 ± 1.15^b^	3.11 ± 0.25^b^	1.28 ± 0.22^b^	108.25 ± 2.15^b^
**APHM**	300	32.65 ± 1.22^b^	2.85 ± 0.21^b^	1.35 ± 0.13^b^	102.22 ± 2.25^b^
**Silymarin**	100	18.10 ± 1.75^b^	4.11 ± 0.15^a^	1.55 ± 0.20^b^	110.54 ± 2.10^b^

Naïve groups did not received any treatment while control received CCl_4_ only

*n* = 6, Average values ± SEM,

^a^*p* ≤ 0.05 significant as compared to control, ^b^*p* ≤ 0.005 highly significant as compared to control

^c^Reduced glutathione, ^d^Thiobarbituric acid reactive substance, ^e^*Ajuga parviflora* hydromethanolic extract

### Effect of APHM on renal histoarchitecture

**Fig. 2:**
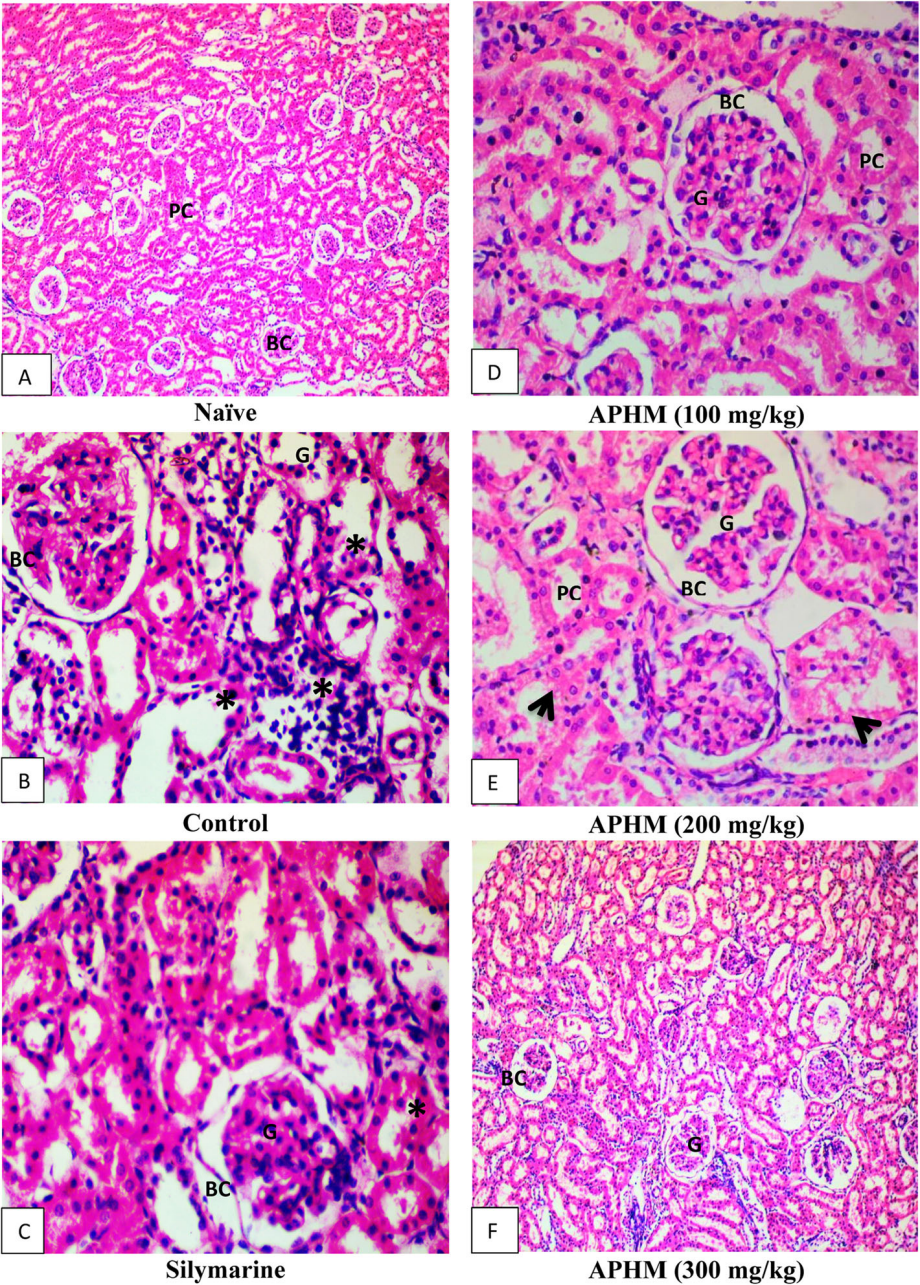
Histopathology of renal tissue of *A. parviflora* at 40× magnification in rat treated with CCl_4_. APHM = *Ajuga parviflora* hydromethanolic extract, BC = bowman capsule, G = glomerulus, PC = proximal convoluted tubules. Arrow head show damaged proximal tubules, steric shows loss of brush boarder

**Fig. 3:**
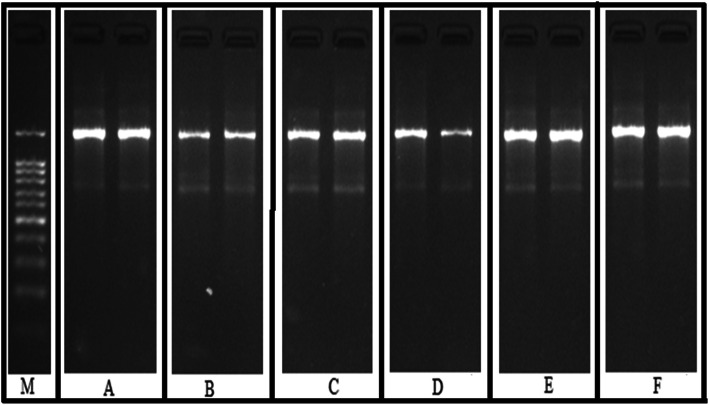
Agarose gel showes CCl_4_ induced DNA damaged of kidney and protective effect of *A. parviflora* in different experimental groups of rats. M = Loading control, A = naïve, B = control, C = Silymarin, D = APHM (100 mg/kg), E = APHM (200 mg/kg), F = APHM (300 mg/kg). The samples were derive from the same experiment and that gels were processed in parallel

The histological features of animals included in this study are highlighted in Table 8. The histological study revealed that the control animals encountered major histological changes like tubular, glomerular and blood vessel congestions. The inflammatory cells and epithelial cells shading were also observed in the control group. However, *A. parviflora* (200 and 300 mg/kg) administration efficiently reversed the CCl_4_ glomerular, tubular and blood vessel congestion. The inflammatory cells and epithelial desquamation were also not observed at 300 mg/kg administration of *A. parviflora*. The histological changes are also presented in Fig. 2A-F. The inflammatory cell or the pus cells are shown with asteric and the damaged epithelial linings are traced with an arrowhead in the control group. After treatment with *A. parviflora,* significant recovery of necrotic tissue with diminished inflammatory cells, proximal tubules, distill convoluted tubules and blood vessels was recorded. At the dose of 300 mg/kg, *A. parviflora* extract showed significant healing in the necrotic and damaged tissue. The Bowman capsule shapes were reversed with normal space and no congestion was observed on blood vessels of the glomerulus. In addition, significant improvement was observed in the CCl_4_ induced DNA damage of kidney after treatment with *A. parviflora* in dose dependent manner as shown in Fig. 3.

**Table 8:** Effect of APHM on renal histoarchitecture after CCl_4_ induced renal injury in rats

Treatment Groups	Doses (mg/kg)	Tubular congestion	Tubular cast	Glomerular congestion	Blood vessel congestion	Inflammatory cells	Epithelial Desquamation
**Naïve**	–	–	–	–	–	–	–
**Control**	–	+++	+++	+++	++	+++	+++
**APHM**^a^	100	++	+	++	+	+	++
**APHM**	200	+	+	+	+	–	+
**APHM**	300	+	–	–	–	–	–
**Silymarin**	100	++	+	++	–	+	+

Naïve groups did not received any treatment while control received CCl_4_ only

*n* = 6,

(+) low (++) moderate (+++) severe presence of histological abnormality while (−) indicates absence of histological abnormalities

^a^*Ajuga parviflora* hydromethanolic extract

## Discussion

In the current study, the phytochemical analysis revealed the presence of important phytochemical constituents, along with biologically potent short-chain polypeptides. The important pharmacological constituents such as vitamin E, vitamin C, stigmasterol and polyphenolic compounds in *A. parviflora* leaf ethanolic extract were previously reported using Soxhlet extraction method [14]. Similarly, tannins, alkaloids, flavonoids, saponins, phenols and polypeptides were reported in *A. parviflora* leaf methanolic extract by Yousaf et al. in 2018 using same Soxhlet extraction method [10]. The estimation of LC_50_ of medicinal plants is an essential and valuable criterion for the presence of toxic constituents. The LC_50_ results of *A. parviflora* obtained in brine shrimp lethality assay suggested that *A. parviflora* leaf extract can be possibly enriched with different important bioactive compounds. The extract of *A. parviflora* did not achieve LC_50_ at 1000 μg/mL against brine shrimps, while etopside showed 100% lethality at 10 μg/mL. Etoposide is used to treat certain forms of cancers and produced significant cytotoxic effects on living cells. Etoposide works by slowing the growth of cancer cells. However, researchers reported that dimethyl sulfoxide (DMSO) is a safer solvent in brine shrimp lethality bioassay even at its highest concentration. Hence, in this study, etoposide and DMSO were utilized as standard and negative control respectively. This broad difference in lethal concentration between the test sample and standard etoposide suggests that *A. parviflora* is pretty safe for normal body cells. Moreover, these values of LC_50_ for plant extract lie under the toxicity profile of non-toxic according to Clarkson’s toxicity criterion for the brine shrimp test. The reported studies suggest that there is a significant correlation between LC_50_ of brine shrimp’s lethality assay and LD_50_ of acute oral toxicity in mice [37]. The acute toxicity results of APHM extract were appreciable at a higher dose i.e. 5000 mg/kg. The results showed an additional characteristic in tested animals like hair loss, which may be due to the presence of anticancer compounds in the extract as found in phytochemical analysis. Therefore, the extract needs further investigation for its safety in some advanced research models.

Free radicals are the major cause of both acute and cronic complications, and in turn lead to diverse morbidities [38]. Free radicals are mainly involved in DNA damages, lipid peroxidation and protein injuries. A kidney is the most vulnerable organ that is prone to the development of acute and chronic renal failure due to free radicals and reactive oxygen species [39]. CCl_4_ administration to animals in this study altered the oxidant-antioxidant balance. The oxidant-antioxidant balance is maintained by the antioxidant enzymes deface system, which is shifted toward negative after CCl_4_ administration. The nephrotoxic effect due to free radical’s generation after CCl_4_ administration has been previously proved in various studies [40, 41]. Several studies validated urine profile as a documented evidence for kidney physiological functions. The kidney distressed conditions are depicted in altered urine profiles. The elevated enzymes and evidence of RBCs, epithelial and pus cells in urine are the signs of glomerular damages. The marked deviations were noticed in the physiological profile of urine in CCl_4_ treated rats. Free radicals generations after CCl_4_ administration may be responsible for such changes in renal tissue. In present study few parameters such as RBCs, pus cells, albumin and epithelial cells were ameliorated, while pH was decreased as compared to the naïve group. These changes in biochemical parameters post CCl_4_ intoxication provide information related to the kidney and liver along with their acid-base balance in the body. Under normal circumstances, urobilinogen is not excreted/ or very little in urine, but due to renal intoxication after CCl_4_ administration the level of urobilinogen was significantly increased. Similarly, augmented enzyme levels were observed after CCl_4_ administration, which reflects diminished renal blood flow due to arterial stenosis accompanied by proteinuria and dehydration. The abnormally increased level of proteinuria may be linked to a severely damaged nephron due to CCl_4_ administration. Under normal circumstances, low molecular weight proteins after filtration are reabsorbed and metabolized by distal tubules [42]. The reversal of these parameters from their deviated levels was bottomed to normal physiological levels in *A. parviflora* treated groups. Our findings were very much similar to previous studies that reported the nephroprotective activity of other plant extracts showing significant restoration of urine profile, antioxidant enzymes, lipid peroxidation, and kidney histopathology [26, 40, 42].

In addition, CCl_4_ induced kidney toxicity was also reflected in serum profile, where impaired renal biochemical values were noticed in blood serum. The increased levels of creatinine, urobilinogen and urea were noticed in rat serum, while protein and albumin levels were considerably reduced, indicating CCl_4_ intoxicated kidney [43]. The increased level of serum creatinine indicates severe renal injury. The outcomes of this study are streamlined with the reported literature [40, 44], which claimed a significant increase in urea, urobilinogen, and creatinine levels in CCl_4_ intoxicated rats as compared to the naïve group. However, the altered serum biochemical parameters were reversed with *A. parviflora* treatment, probably due to the protective effect against CCl_4_ induced renal damages.

In the current study hydrogen peroxide and nitrites concentrations were markedly elevated in CCl_4_ treated group and might be due to the oxidative stress. Nitrites generate nitric oxide under an acidic condition which is converted to peroxynitrite with the help of superoxide, a highly damaging molecule involved in tissue damage. However, *A. parviflora* co-administration reversed the elevated level of nitrites. These findings are in good agreement with the results of the previous studies where the levels of nitrites were decreased by the plant extract administration in animals having CCl_4_ induced nephrotoxicity [43, 45].

The intrinsic cellular antioxidant enzymes i.e. phase I and phase II play a key role in the cellular defense system and are responsible for the elimination of induced oxidative stress. Specifically, H_2_O_2_ levels become elevated in kidney due to the oxidative stress induced by CCl_4_. Similarly, superoxides are also catalyzed to H_2_O_2_, which will be further catalyzed to water and oxygen. The diminished phase I antioxidant enzymes in CCl_4_ treated group is linked to elevated H_2_O_2_ while co-administration of *A. parviflora* ameliorates the levels of antioxidant enzymes. The improved level of anti-oxidant enzymes justifies the kidney repairing effect of *A. parviflora* from pathological conditions. However, the protective effects of *A. parviflora* were dose dependent like 300 mg/kg body weight showed noticeable results as compared to 100 and 200 mg/kg body weight dose. Usually, plant extracts with polyphenolic compounds are previously reported with a similar protective effect in CCl_4_ intoxicated rats [46]. The phase II enzymes i.e. GST and GSR were also considered for examination in CCl_4_ fed rats. The reduced levels of phase II enzymes reflect renal tissue damaged due to oxidative stress. CCl_4_ intoxication is responsible for the generation of lipid peroxides which as a result not only depressed the activity of these enzymes but also reduced GSH concentration [47]. With the help of GSH, glutathione peroxidase removed major free radicals like hydrogen peroxide and other hydroperoxides from the environment. On the other hand, glutathione-S-transferase plays a key role in the conjugation of toxicants with glutathione.

The biochemical outcomes of this study were further endorsed via microscopic investigations of histopathology. The notable histological modifications were observed in different groups of animals according to their treatments. In the animals of the CCl_4_ treated group, the kidney demonstrated loss of Bowman’s capsule wall and severe epithelial lining damage of collecting tubules with tissue necrosis. The changes in tissue may be as a result of free radical generation after CCl_4_ administration. Previous studies also reported such classical damages in rat kidneys after CCl_4_ administration, such as loss of the epithelial lining of convoluted tubules, brush border loss and tissue necrosis [46]. The reverse of such destructed changes and regain of normal anatomical features were in accordance with post administration of *A. parviflora* in dose dependent manner. Our results were following the various reported histopathological findings by Khan et al. on methanolic extract of *Citharexylum spinosum* [46]. The antioxidant and therapeutic effects were relating to polyphenolic constituents of *Citharexylum spinosum*. Moreover, DNA in this study was significantly damaged in the CCl_4_ treated group as compared to naïve animals. The frequent generations of reactive species such as lipid peroxidase that caused genetic mutation via strand breakdown [48–50]. The enhanced level of oxidative stress directly escalates Deoxy-ribonuclease I activity along with DNA damage via base-pair modification [50]. Contrarily, co-administration of *A. parviflora* reduced DNA damage in dose dependent manner in rat renal tissue possibly by reducing oxidative stress and Deoxy-ribonuclease I activity. These findings were streamlined with Khan et al. [40]. However, further studies are still needed to illustrate exact mechanism on molecular basis for these potential therapeutic activities of plant extract.

## Conclusion

Cumulatively the data gathered in this study suggested the presence of various pharmacologically active constituents in APHM leaves extract. The findings of the current study concluded that *A. parviflora* is rich with bioactive compounds that can effectively improve nephrotoxicity induced by CCl_4_. However, further studies are needed to isolate and structurally elucidate the bioactive compounds of *A. parviflora* responsible for its nephroprotective activity.

## Supplementary Information

**Additional file 1.**

## Data Availability

All data generated or analyzed during this study are included in this published article.

## Abbreviations

CCl4: Carbon tetra chloride
LC/MS: Liquid chromatography mass spectroscopy
LD: Lethal dose
ARF: Acute renal failure
TCA: Trichloroacetic acid
TBARS: Thiobarbituric acid reactive substance
TRAP: Telomerase repeated amplification protocol
