# Supplementary figures and images for: Phytochemical, acute toxicity and renal protective appraisal of Ajuga parviflora hydromethanolic leaf extract against CCl4 induced renal injury in rats
Source: BMC Complement Med Ther. 2021 Jul 12;21:198. doi: 10.1186/s12906-021-03360-9 (PMC8276434; doi:10.1186/s12906-021-03360-9)

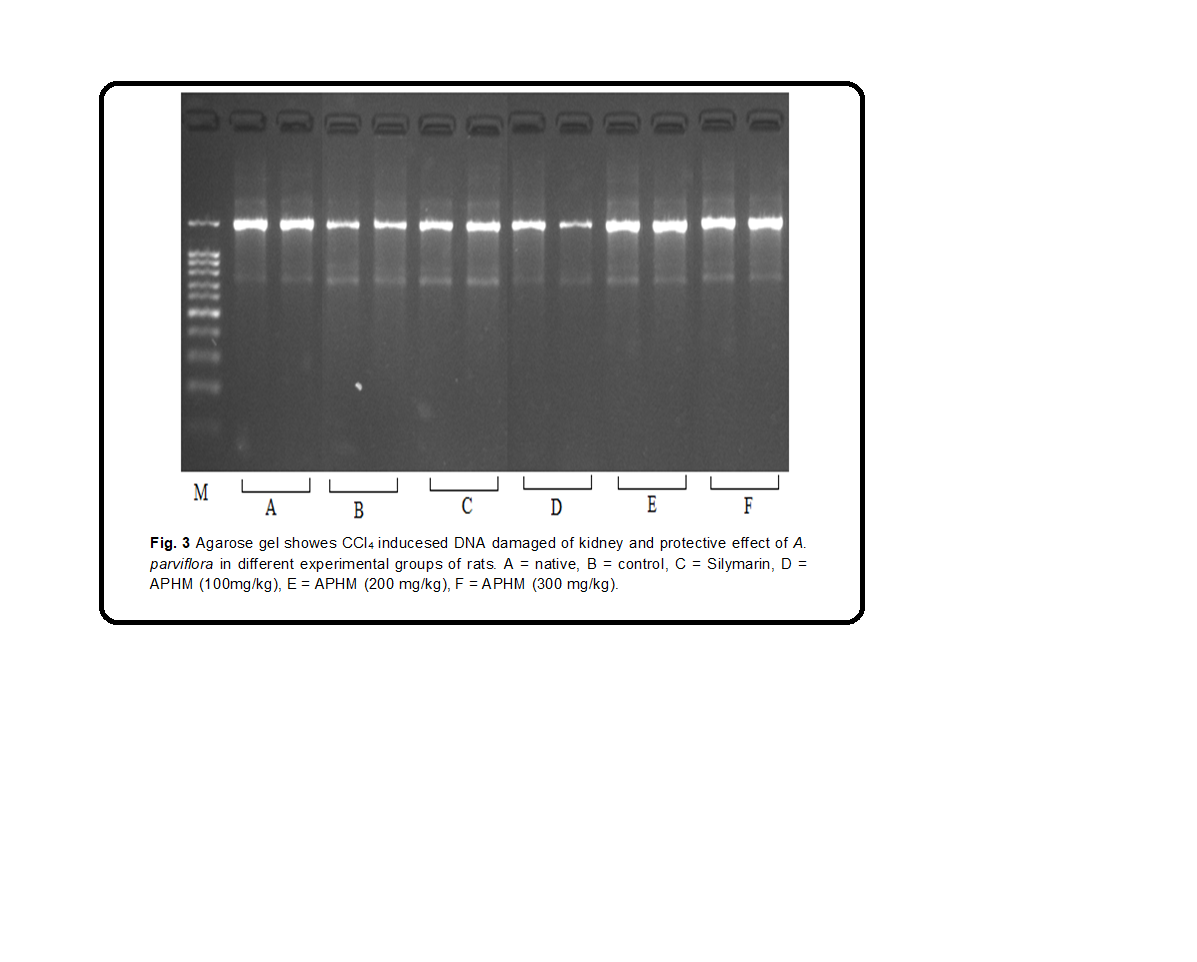


**Original and unprocessed versions of PCR images**

Supplement: Supplementary file 1 — Additional file 1. [file 12906_2021_3360_MOESM1_ESM.docx]
